# Promoter Methylation-Mediated NPTX2 Silencing Promotes Tumor Growth in Human Prostate Cancer

**DOI:** 10.7150/jca.65214

**Published:** 2022-01-01

**Authors:** Yixi Su, Qiang Huang, Li Lu, Hu Qu, Dejuan Wang, Jianguang Qiu, Weiqian Li, Mengmeng Lin, Huanliang Liu, Zhongyang Wang, Xiangling Yang

**Affiliations:** 1 Department of Clinical Laboratory, The Sixth Affiliated Hospital, Sun Yat-sen University, Guangzhou, Guangdong, China.; 2 Guangdong Provincial Key Laboratory of Colorectal and Pelvic Floor Diseases, Guangdong Institute of Gastroenterology, The Sixth Affiliated Hospital, Sun Yat-sen University, Guangzhou, Guangdong, China.; 3 Department of Urology, The Sixth Affiliated Hospital, Sun Yat-sen University, Guangzhou, Guangdong, China.

**Keywords:** cancer targeted therapy, DNA methylation, demethylation, NPTX2, prostate cancer

## Abstract

Neuronal pentraxin 2 (NPTX2), a secretory protein of neuronal pentraxins, was first identified in the nervous system. Several studies have shown that expression levels of NPTX2 are associated with the development of various cancers. However, whether NPTX2 is involved in prostate cancer progression is unclear. Herein, we found that NPTX2 is significantly reduced in prostate cancer tissues and cancer cell lines compared to control prostate tissues and control prostatic epithelial cell lines. Furthermore, the NPTX2 promoter is highly methylated in prostate cancer cells. Consistently, NPTX2 could be restored by treatment with the DNA methyltransferase inhibitor 5-aza-2′-deoxycytidine (decitabine, 5-AZA-dC). Overexpression of NPTX2 inhibited prostate cancer cell proliferation both *in vitro* and *in vivo*. In conclusion, our study demonstrated that NPTX2 acts as a tumor suppressor gene in prostate cancer.

## Introduction

Prostate cancer is the fourth most commonly diagnosed cancer worldwide, according to the latest statistics for 2020 from the International Agency for Research on Cancer 1, 2. Although androgen deprivation therapy (ADT) is effective during the initial stage, patients invariably progress from the androgen-sensitive stage to castration-resistant prostate cancer (CRPC) 3, 4. Once men suffer from CRPC, poor prognosis is inevitable despite some available chemotherapies, such as docetaxel, enzalutamide, and abraterone 5-8. Therefore, there is an urgent need to explore the mechanism underlying prostate cancer.

Growing evidence indicates a close relationship between the risk of cancer and neurological diseases 9, 10. Neuronal pentraxin 2 (NPTX2), a secretory protein of neuronal pentraxins, was first identified in the nervous system. It promotes the formation of excitatory synapses and plays a significant role in the process of synaptic remodeling 11, 12. NPTX2 is widely distributed in the brain, heart, liver, pancreas, testis and skeletal muscle 13. Increasing evidence demonstrates that NPTX2 acts as either an oncogene or tumor suppressor in different tumor types, being significantly upregulated in colorectal cancer (CRC) 14 and frequently downregulated in pancreatic cancer 15 compared to control tissue. Higher NPTX2 expression levels accelerate the progression of CRC and indicate poorer CRC prognosis 16. Other studies have shown that NPTX2 is frequently downregulated in pancreatic cancer and contributes to the proliferation and invasion of pancreatic cancer cells 15, 17. Collectively, these findings indicate that NPTX2 plays distinct roles in different malignancies. In this study, we aimed to explore the role and potential mechanism of NPTX2 in prostate cancer.

## Materials and Methods

### Cell Lines and Cell Culture

The human prostate cancer cell lines C4-2, LNCaP, DU145, and PC3 and the control prostatic epithelial cell line RWPE-1 were purchased from American Type Culture Collection (ATCC). Human prostate cancer cell lines were cultured in RPMI 1640 medium (Gibco, China) supplemented with 10% fetal bovine serum (FBS) and 1% penicillin and streptomycin (PS). RWPE-1 cells were cultured in keratinocyte serum free (KM) (Gibco, US) with 0.5 mg/ml bovine pituitary extract (BPE) and 5 ng/ml human recombinant epidermal growth factor (EGF). All cells were incubated at 37°C with an atmosphere of 5% CO_2_. 5-Aza-2′-deoxycytidine (decitabine, 5-AZA-dC, Sigma, US) powder was dissolved in DMSO.

### Lentivirus Transfection

Lentiviruses carrying NPTX2 cDNA and vector control (VC), which were packaged by Vigene Biosciences (US), were transfected into DU145 and PC3 cells following the manufacturer's instructions to construct NPTX2-overexpressing cell lines and VC. Stable cell lines were maintained in low-concentration (1 μg/mL) puromycin after two weeks of 10 μg/mL puromycin selection.

### Western Blot (WB)

Radioimmunoprecipitation assay (RIPA) buffer (Cell Signaling Technology, US) supplemented with protease inhibitor and phosphatase inhibitor (Selleck, US) was used to collect cellular proteins. Then, the protein concentration was evaluated using the bicinchoninic acid (BCA) assay (Thermo Fisher Scientific, US). SDS-polyacrylamide gel electrophoresis assay and transfer to PVDF membranes were performed followed by incubation for an hour in blocking buffer containing 5% skimmed milk. Next, primary antibodies were incubated overnight at 4℃. The next day, after incubation with HRP-conjugated rabbit or mouse secondary antibody (Santa Cruz Biotechnology, US) for 2 hours at room temperature, chemiluminescence reagent was used, and blots were visualized using a chemiluminescence imager (Bio-Rad, US) and analyzed using Image Lab Software (Bio-Rad, US) or physical film in the darkroom. The antibodies used were NPTX2 (Cat. No. 10889-1-AP, Proteintech, the US) and GAPDH (Cat. No. 10494-1-AP, Proteintech, the US).

### DNA Isolation and Methylation Assays

Genomic DNA was extracted from cells lines using a DNA-Tissue Kit (Omega, US). Then, the obtained DNA was treated with a bisulfite conversion kit (TIANGEN, China), which converts unmethylated cytosines from a DNA sample into uracils while methylated cytosines remained unchanged. Based on this principle, MethPrimer (http://www.urogene.org/cgi-bin/methprimer/methprimer.cgi) was used to design primers for the original sequence of the NPTX2 promoter (NCBI) fragment and a fragment that replaced cytosines with uracils. The methylated primer pairs (M) were as follows: forward, 5'- ATCGTCGTGTAGTAGAAGGAGAC-3'; reverse, 5'-TATCCTTACCCGTAACCCCC -3', while the nonmethylated primer pairs (U) were forward, 5'-AGATTGTTGTGTAGTAGAAGGAGAT-3'; reverse, 5'-ATAATATCCTTACCCATAACCCCC-3'. Methylation-specific PCR (MSP) was performed using a methylation-specific PCR kit (TIANGEN, China).

### DNA Analysis by Agarose Gel Electrophoresis Assay

According to the molecular weight of the separated DNA, a gel with the corresponding concentration was prepared. The solvent for the gel was 0.5×Tris-borate-EDTA (TBE), and the solute was agarose powder. GelRed (Solarbio, China) was added to the gel at a ratio of 1:10,000. DNA samples (10 ~ 20 μL) that were previously mixed with DNA gel loading dye (Thermo Scientific, US) were added to the gel, and electrophoresis was performed. The strips were visualized using a chemiluminescence imager (Bio-Rad, US) and analyzed using Image Lab Software (Bio-Rad, US).

### RNA Isolation and Real-Time Quantitative Polymerase Chain Reaction (RT-QPCR)

Total RNA was isolated using an RNA-Quick Purification Kit (YISHAN, China) and then reverse transcribed to cDNA by a PrimeScript™ RT reagent kit (TakaRa, China). Real-time PCR was performed using a SYBR Green Premix Ex Taq II Kit (TaKaRa, China) in an ABI ViiA7 fast real-time PCR system. The PCR procedure was set as follows: initial denaturation at 95°C for 30 seconds, followed by 40 cycles of 95°C for 5 seconds and 60°C for 30 seconds, followed by a final elongation step at 95°C for 15 seconds, 60°C for 60 seconds, and 95°C for 15 seconds. The relative mRNA expression of NPTX2 was calculated using the 2^-△△Ct^ method relative to GAPDH mRNA expression. The specific primers for the NPTX2 promoter were as follows: forward, 5'-GCCAACGAGATCGTGCTGAT-3', reverse: 5'-TTGCCGTCACTGACAAACAG-3'. The specific primers for GAPDH were as follows: forward: 5'-GCACCGTCAAGGCTGAGAAC-3', reverse: 5'-TGGTGAAGACGCCAGTGGA-3'.

### Immunohistochemistry (IHC)

Subcutaneous tumors from nude mice were sectioned in paraffin. The sections were deparaffinized in xylene (twice, 10 min each) and rehydrated in a series of concentrations of ethanol (95%-70%, 3 mins each). Endogenous peroxidase activity was blocked using 0.3% H2O2 (diluted in ddH2O) for 10 minutes. Then, antigen retrieval was performed in a microwave with tissues incubated in citrate buffer (pH = 6.0) (refer to the instructions of the primary antibody for specific retrieval conditions). Sections were blocked in 5% goat serum (BOSTER, US) for 30 mins at room temperature and then incubated with primary antibody against NPTX2 (Cat. No. 10889-1-AP, Proteintech, the US) and Ki67 (Cat. No. 12202S, Cell Signaling Technology, US) overnight at 4 ℃. Secondary antibody (Dako, Denmark) was incubated for 1 h at room temperature, and the signal was subsequently detected using a chromogenic substrate (Dako, Denmark). Finally, the sections were counterstained with hematoxylin and then imaged under a Leica DM1000 LED Microscope (Leica Microsystems, Germany) using the same light source and the same image parameters at appropriate magnification.

The evaluation criteria for NPTX2 and Ki67 protein levels included assessing their average optical density values (AODs). The specific process involved selecting tumor sections from 5 mice in each group and randomly selecting 5 fields from each section at 20× magnification. ImageJ was used to calculate the integrated optical density (IOD) of the DAB-positive area and the area of tissue in the field. The latter was divided by the former to obtain the AOD of positive signals in the field.

### Cell Proliferation Assay

The xCELLigence system (Roche Applied Science, Germany) was utilized to dynamically monitor cell proliferation. Briefly, cells in 100 μL of medium were seeded into E-plates. The measurable impedance increased when cells proliferated. The impedance is expressed as the normalized cell index, an arbitrary unit. Measurements were analyzed using the Real-Time Cell Analysis (RTCA) software supplied by the manufacturer.

### Colony Formation Assay

NPTX2-overexpressing DU145 and PC3 cells constructed using the methods mentioned above were used for colony formation, and cells were seeded into six-well plates at 500 cells/well and cultured for 8-9 days. After fixing in ice-cold methanol and staining with Coomassie brilliant blue, images were obtained to quantify the colonies.

### Mouse Xenograft Model

To establish a xenograft model, 5×10^6^ DU145 cells were subcutaneously injected into the flank of each 6-week-old male BALB/c-nu mouse (Nanjing Biomedical Research Institute of Nanjing University). Tumor volumes were measured using standard calipers and calculated using the following formula: L (length)×W (width)^2^×0.5. At the end of the study, the tumors from sacrificed mice were removed, weighed and stored for further experiments. All animal studies were conducted with the approval of the Institutional Animal Care and Use Committee of Guangdong Laboratory Animals Monitoring Institute.

### Statistical Analysis

Data were analyzed using GraphPad Prism (version 7.04) and Excel. Differences between means were evaluated using Student's t test or ANOVA, and p < 0.05 was considered statistically significant.

## Results

### NPTX2 expression is reduced in prostate cancer

**Figure 1:**
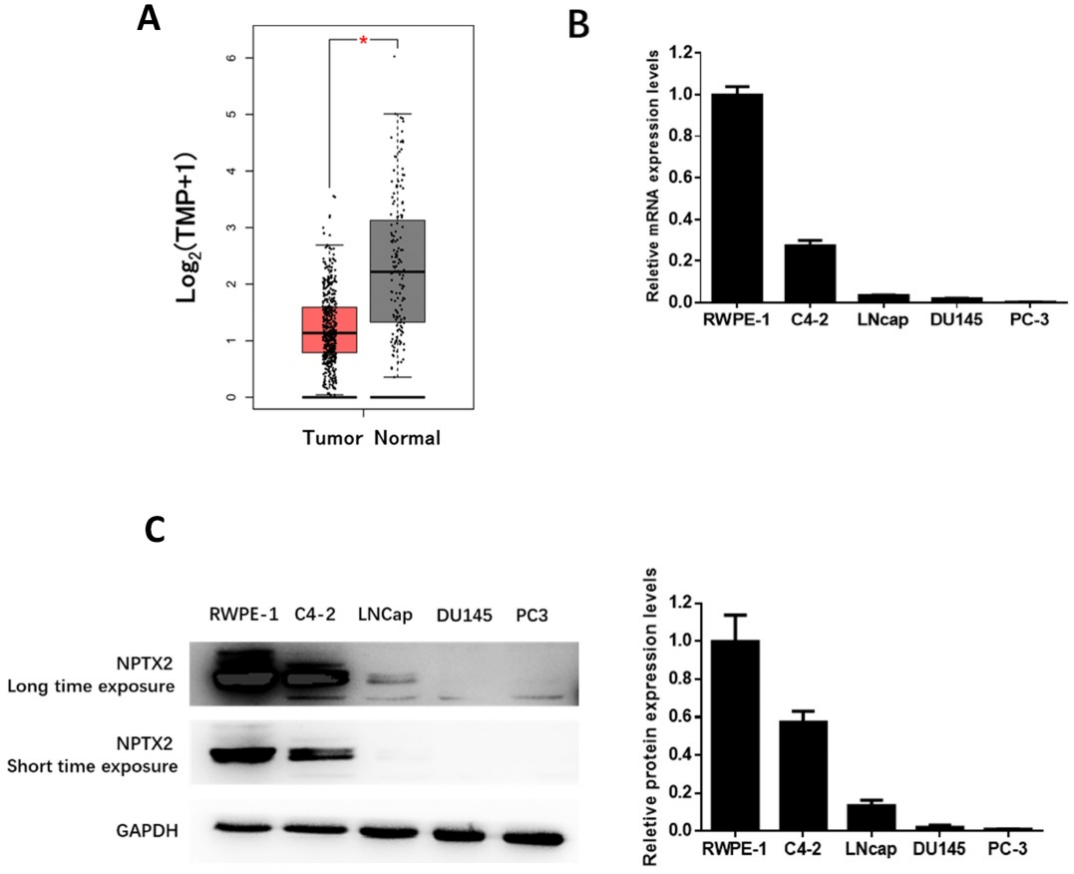
** NPTX2 expression was reduced in prostate cancer.** (A) NPTX2 mRNA was significantly reduced in prostate cancer tissues compared to normal control in match TCGA normal using the GEPIA online database. (B) QPCR analysis of NPTX2 mRNA expression levels in prostate cancer cell lines (C4-2, LNcap, DU145, PC3) and normal prostatic epithelial cell RWPE-1. (C) Western blot analysis of NPTX2 protein expression levels in prostate cancer cell lines (C4-2, LNcap, DU145, PC3) and normal prostatic epithelial cell RWPE-1.

To examine the role of NPTX2 in the tumorigenesis and progression of prostate cancer, we searched The Cancer Genome Atlas (TCGA) cancer pandataset obtained from the GEPIA online database 18. The inclusion and exclusion criteria of prostate cancer in the TCGA database (Table S1) and the detailed clinical parameters of the enrolled patients (Table S2) are shown in the Supplementary Materials 19. Additionally, the detailed clinical parameters of the enrolled patients are shown in TCGA database, which demonstrated that the expression of NPTX2 in prostate cancer was significantly lower than that in control tissues (p < 0.05, Figure 1A). Furthermore, we performed qPCR and western blot assays to determine the expression of NPTX2 in several prostate cancer cell lines, C4-2, LNCaP, DU145, and PC3, and the control prostatic epithelial cell line RWPE-1. The results showed that expression of NPTX2 in control cells was higher than that in several prostate cancer cell lines. Interestingly, NPTX2 levels in C4-2 cells were higher than those in LNCaP, DU145 and PC3 cells, while NPTX2 was almost absent in the latter three cell lines (Figure 1B, 1C).

### Promoter hypermethylation mediates the silencing of NPTX2 in prostate cancer

**Figure 2:**
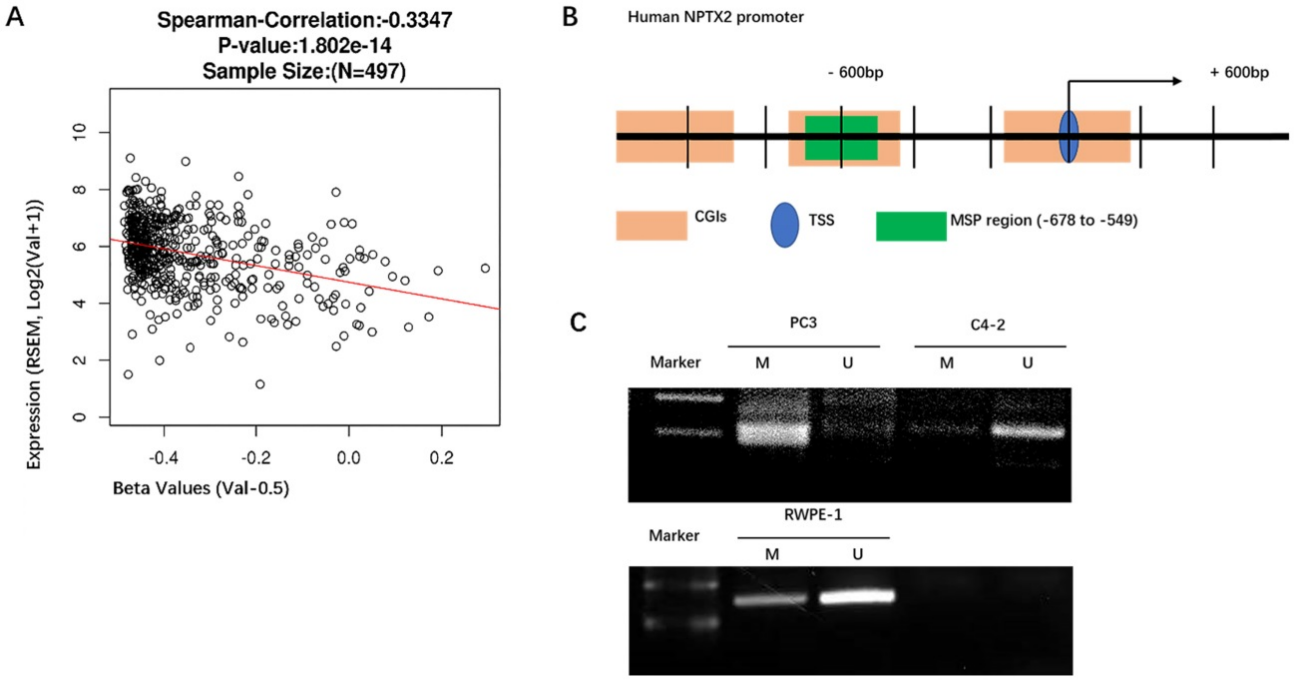
** Promoter hypermethylation mediated the silence of NPTX2 in prostate cancer.** (A)Spearman correlation test of NPTX2 methylation status with NPTX2 mRNA expression in TCGA PRAD cohort using the linkedOmics online database. (B) NPTX2 promoter sequence from NCBI, A sequence fragment (-678bp~-549bp) highly concentrated in CpG islands was selected to conduct methylation specific PCR (MSP). (C) Methylation assay of the NPTX2 promoter in RWPE-1 and PC3 cells. The products were separated electrophoretically on 2% agarose gels. M, amplification with methylated primers; U, amplification with unmethylated primers.

Because NPTX2 has been reported to be highly methylated in pancreatic cancer, we analyzed the methylation levels of NPTX2 in a TCGA cancer pandataset obtained from the linkedOmics online database (http://www.linkedomics.org). NPTX2 mRNA expression levels were negatively correlated with NPTX2 promoter methylation in a cohort of prostate cancer patients in the TCGA Research Network (n = 497). With the increase in NPTX2 promoter methylation, expression levels of NPTX2 mRNA decreased (r = -0.349, p < 0.001, Figure 2A). The NPTX2 promoter sequence was downloaded from NCBI, and MethPrimer (http://www.urogene.org/cgi-bin/methprimer/methprimer.cgi) was used to predict CpG islands. A sequence fragment (-678 bp ~ -549 bp) highly concentrated in CpG islands was selected to conduct methylation-specific PCR (MSP) (Figure 2B), which was performed in RWPE-1 and PC3 cells, and the details of the steps are shown in the *Materials and Methods*. The amplified products of MSP were analyzed using DNA gel electrophoresis. This result was consistent with the RNA and protein results. NPTX2 was highly methylated in PC3 cells and not methylated in RWPE-1 cells (Figure 2C).

### The demethylating agent 5-aza-2′-deoxycytidine restores NPTX2 expression in prostate cancer cell lines

**Figure 3:**
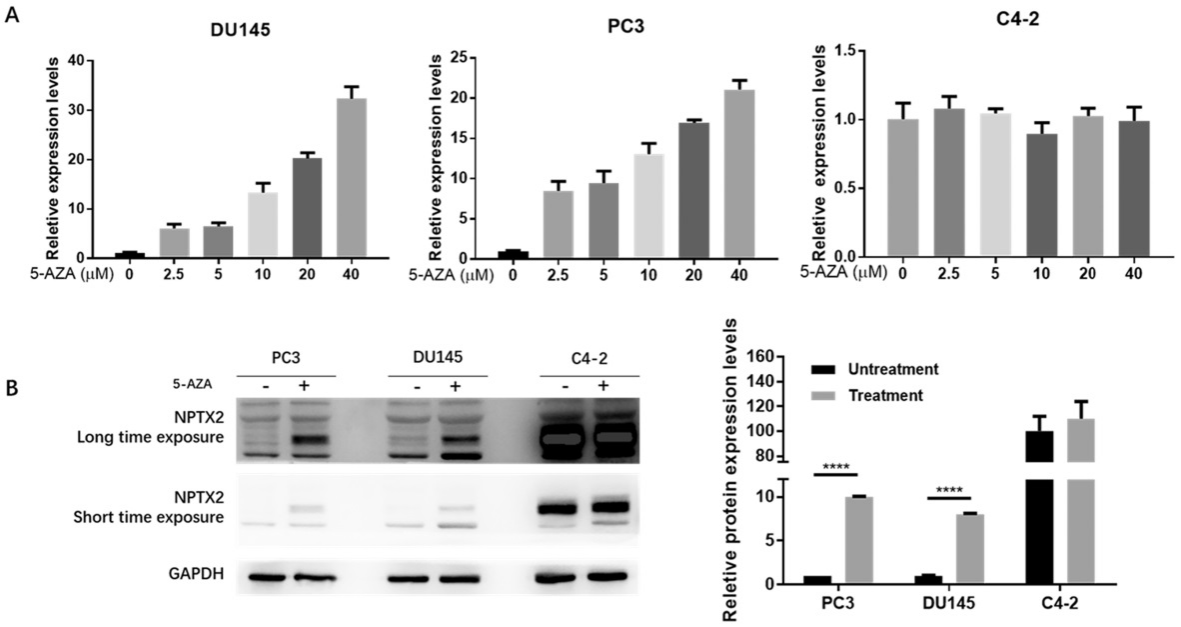
** Demethylation agent 5-Aza-2′-deoxycytidine restored NPTX2 expression in DU145 and PC3 cells.** (A) QPCR analysis of NPTX2 expression in DU145, PC3 and C4-2 cells with or without the treatment of 5-AZA-dC for 72h. (B) Western Blot analysis of NPTX2 expression in DU145 and PC3 cells with or without the treatment of 40µM 5-AZA-dC for 48h (****p < 0.0001).

To further confirm that the low expression of NPTX2 in prostate cancer is due to promoter methylation, we treated DU145, PC3 and C4-2 cell lines with the demethylating agent 5-aza-2′-deoxycytidine (5-AZA-dC). To explore the optimal experimental conditions, we used six drug concentration gradients: 0 (no treatment control), 2.5, 5, 10, 20 and 40 μM. qPCR results showed that 40 μM was the optimal 5-AZA-dC treatment concentration (treated for 72 h). As shown in Figure 3A, the relative mRNA expression of NPTX2 increased with increasing 5-AZA-dC concentration in DU145 and PC3 cells but caused no significant change in C4-2 cells. Furthermore, WB results showed that NPTX2 protein levels in the 5-AZA-dC treatment group (40 μM for 48 h) were significantly higher than those in the control group. Consistently, expression of NPTX2 in C4-2 cells was not significantly different from that in the 5-AZA-dC-free group and was much higher than that in DU145 and PC3 cells (Figure 3B). This evidence suggested that the NPTX2 promoter is methylated and that its expression is inhibited in prostate cancer cells.

### Overexpression of NPTX2 inhibits prostate tumor growth *in vitro*

**Figure 4:**
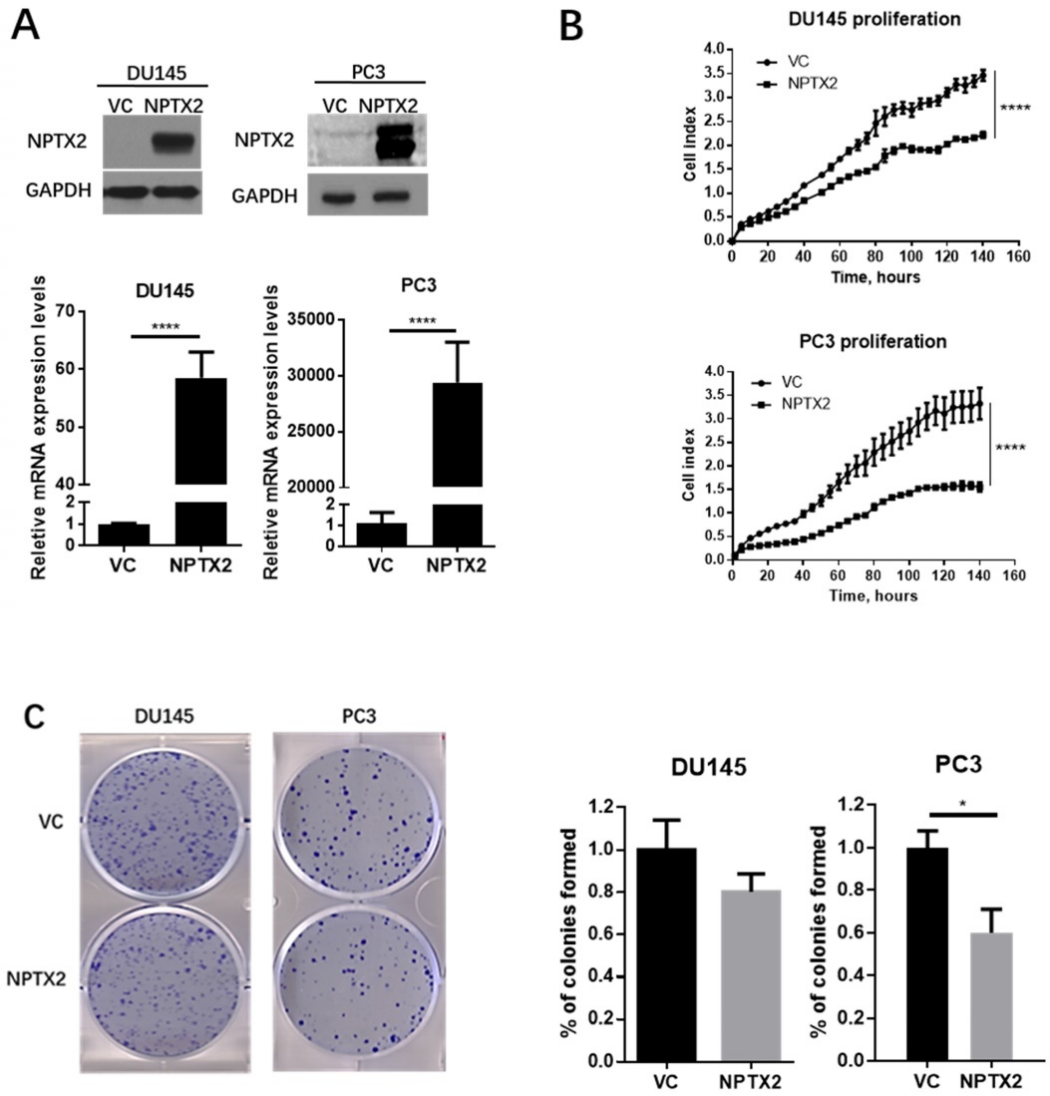
** NPTX2 suppresses prostate cancer cells proliferation, colony formation and tumor spheres formation *in vitro*.** (A) QPCR and western blot confirmed the restored expression of NPTX2 in PC3 and DU145. Statistical significance was determined by a two-tailed, unpaired Student's t-test (****p<0.0001). (B) RTCA analysis of the proliferation ability of DU145 and PC3 cells infected with pLenti-NPTX2 or pLenti-vector Statistical significance was determined by a two-tailed, unpaired Student's t-test (****p<0.0001). (C) Colony formation analysis of DU145 and PC3 cells infected with pLenti-NPTX2 or pLenti-vector (*p<0.05).

To explore the functions of NPTX2 in prostate cancer, real-time cell analysis (RTCA), colony formation assays and tumor sphere formation assays were conducted in DU145 and PC3 cells that were infected with lentivirus containing NPTX2 cDNA or VC. Figure 4A demonstrates the successful transfection and expression of lentivirus in DU145 and PC3 cells. As shown in Figure 4B and C, NPTX2 overexpression inhibited cell proliferation and colony formation.

### Overexpression of NPTX2 inhibits prostate tumor growth *in vivo*

**Figure 5:**
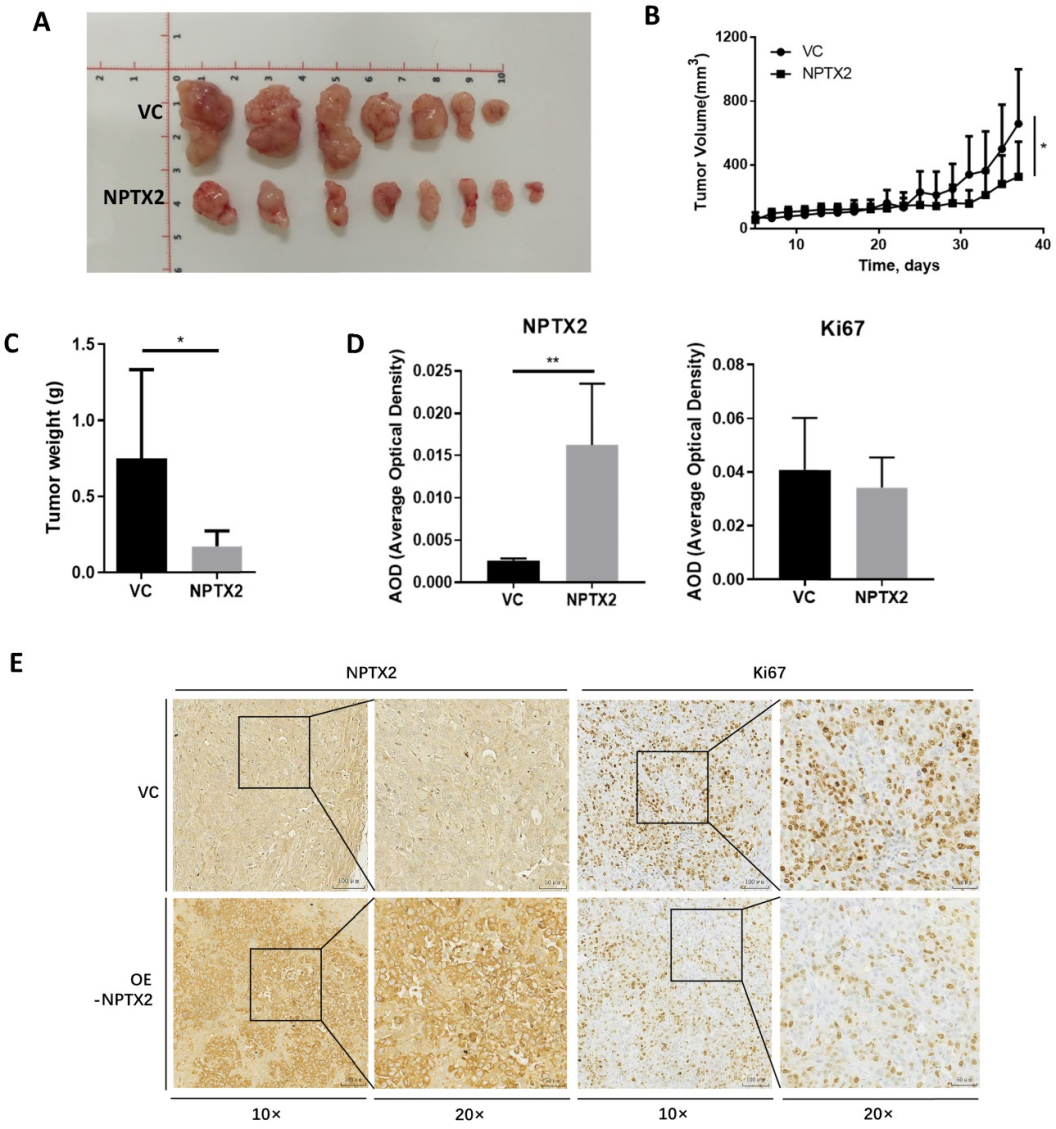
** NPTX2 is associated with prostate cancer progression *in vivo*.** (A) Representative image of prostate cancer xenograft of DU145 cells infected with the pLenti- NPTX2 and the pLenti-Vector. (B) The tumor growth curve of subcutaneous xenograft of DU145 cells with pLenti-NPTX2 or pLenti-vector. Statistical significance was determined by one-way ANOVA (*p<0.05). (C) The tumor weight of DU145 cells with pLenti-NPTX2 or pLenti-vector. Statistical significance was determined by a two-tailed, unpaired Student's t-test (*p<0.05). (D) The average optical density of IHC staining of NPTX2 and Ki67 in sections of xenograft tumor tissues from pLenti- NPTX2 group and the pLenti-Vector group(**p<0.01). (E) Representative images of IHC staining of NPTX2 and Ki67 in sections of xenograft tumor tissues from pLenti- NPTX2 group and the pLenti-Vector group.

To evaluate the effect of NPTX2 on prostate cancer progression *in vivo*, NPTX2-overexpressing DU145 cells stably constructed using lentivirus infection and DU145 cells transfected with vector control were subcutaneously injected into nude mice. The results showed that tumor size and tumor weight in the NPTX2 overexpression group were significantly lower than those in the control group (Figure 5A, B, C). Immunohistochemical staining revealed that lentivirus infection was successful in the NPTX2 overexpression group, while NPTX2 was highly expressed in DU145 cells (Figure 5D), and there was less positive expression of Ki67 in NPTX2-overexpressing tumors than in control tumors (Figure 5E). These data all suggested that NPTX2 suppresses prostate cancer progression *in vivo*.

## Discussion

NPTX2 is a member of the neuronal pentraxin family that is essential for synapse formation. It is closely associated with central nervous system diseases, such as Parkinson's and Alzheimer's diseases 12. Previous studies have reported that NPTX2 is reduced in pancreatic cancer tissues and that its downregulation is relevant to poor prognosis in pancreatic cancer 15, 17. Recent studies have found that abnormal upregulation of NPTX2 expression is correlated with proliferation and metastasis in colorectal cancer, clear cell renal cell carcinoma and neuroblastoma 14, 20, 21. The TCGA dataset revealed that expression of NPTX2 is different in distinct types of cancer, and NPTX2 may represent a prognostic marker in renal cancer and endometrial cancer 22. NPTX2 plays distinct roles in different types of cancer. In this study, we focused on prostate cancer, which is the fourth most commonly diagnosed cancer worldwide 2.

Targeted therapy has become a hot topic in cancer research 23-25. Targeting specific proteins associated with the proliferation and migration of tumor cells using specific drugs or monoclonal antibodies can effectively inhibit the development of cancer 26, 27. A certain mutations or modifications of a protein on the surface of the tumor cell or inside cause changes in its expression level or function. Theoretically, drug treatment targeting this protein can reverse the effect of promoting tumor growth. Therefore, identifying a target is especially critical. In our study, the TCGA dataset provided ideas and support for these tenets 22. NPTX2, which is silenced in prostate cancer but highly expressed in control prostate tissues, has great potential research value. By constructing NPTX2-overexpressing cells for colony formation experiments, we found that the formation of tumor spheres was inhibited in the group in which expression of NPTX2 was restored. Consistent with the *in vitro* results, restoring the expression of NPTX2 inhibited prostate tumor growth *in vivo*, demonstrating that NPTX2 is a key inhibitor of tumorigenesis in prostate cancer. Understanding the silencing mechanism of NPTX2 and reversing this low expression status may be of great significance for the treatment of prostate cancer.

Epigenetic modifications include posttranslational modifications to histones, DNA methylation, and RNA editing. DNA methylation plays an important role in many biological processes due to its role in regulating the expression of key regulatory genes 28. It has been reported that promoter methylation is associated with low expression of NPTX2 in pancreatic cancer 15, 17, 29. Therefore, we hypothesized that DNA hypermethylation might be responsible for the downregulation of NPTX2 in prostate cancer. As mentioned in the results section, TCGA data and our experimental studies, MSP, qPCR, western blot, and prostate cancer cells treated with 5-AZA-dC confirmed that NPTX2 expression in prostate cancer tissues was lower than that in control prostate tissues due to promoter methylation. Methylation of the tumor suppressor promoter is a common feature in tumor cells 30, 31. It is important to improve expression levels of NPTX2 in prostate cancer, and the most straightforward approach for doing this is to remove promoter methylation. In this study, we explored the optimal concentration of the demethylation agent 5-AZA-dC. Epigenetics involves the inclusion of different modifications in gene expression patterns that do not result in changes to the DNA sequence and are reversible and inherited. Of all the epigenetic mechanisms, DNA methylation is the most common. DNA methyltransferases (DNMTs) are responsible for adding methyl groups to the fifth carbon of cytosine donated by S-adenosine (SAM) to CpG dinucleotides. Abnormal changes in this mechanism, particularly hypermethylation of DNA in gene regulatory regions, are a common feature of cancer 32. For example, glutathione S-transferase Pi 1 (GSTP1) is involved in DNA protection and androgen receptor (AR) in prostate cancer 33.

There have been many studies on the treatment of DNA demethylation in prostate cancer models. For example, Mahanine is a plant-derived alkaloid that inhibits DNMT1 and DNMT3B through the proteasomal degradation pathway. In prostate cancer cell lines, this compound inhibits DNMT activity, reduces RASSF1A promoter methylation and induces re-expression 34. Developing effective dimethylating drugs for cancer is a long-term goal of researchers. For targeted therapy, the target must be stable and avoid the side effects of regulating the target on normal cells 35. Demethylation only regulates genes that are methylated in cancer cells and does not affect unmethylated genes in normal cells. Perhaps the reason for the methylation of this gene in cancer cells has important implications for cancer research. Although our results have demonstrated that NPTX2 is expressed at low levels in prostate cancer and that 5-AZA-dC can upregulate its levels in DU145 and PC3 cell lines, NPTX2 is relatively highly expressed in the C4-2 cell line and 5-AZA-dC has no regulatory effect on it, indicating that the NPTX2 promoter is not methylated at C4-2, indicating there are more mechanisms to explore in the occurrence and development of prostate cancer.

In conclusion, this is the first report showing that NPTX2 expression is epigenetically silenced in prostate cancer due to DNA methylation of the NPTX2 promoter. Furthermore, our results suggested that NPTX2 inhibits prostate cancer tumor growth *in vitro* and *in vivo*. In addition, there is still a lack of targeted therapies that produce a long-term, sustainable response in patients with prostate cancer. We expect our study to help provide new strategies for the treatment of prostate cancer patients.

## Supplementary Material

Supplementary tables.
