# Supplementary material for: Promoter Methylation-Mediated NPTX2 Silencing Promotes Tumor Growth in Human Prostate Cancer
Source: J Cancer. 2022 Jan 1;13(2):706–14. doi: 10.7150/jca.65214 (PMC8771508; doi:10.7150/jca.65214)
Supplement: Supplementary file 1 — Supplementary tables. [file jcav13p0706s1.pdf]

Supplementary materials

Table S1 The inclusion and exclusion criteria of prostate cancer in TCGA database

Inclusion criteria	Exclusion criteria
Informed consent of the patient and approval by the local institution.	No patient informed consent or local authority approval.
The primary disease was prostate adenocarcinoma.	The primary disease was not prostate adenocarcinoma.
Availability of samples meeting standards for quality and quantity that include:<br>(1) No RNA degradation occurred in the samples;<br>(2) Primary tumor with a source of matched normal tissue;<br>(3) Blocks frozen in OCT were made of all tumors and of paired benign tissue when present. A 5 micron section was cut from the top or bottom of the OCT block of tumor cases;<br>(4) Normal samples underwent pathology review, and prostate origin (i.e., no seminal vesicles) and absence of tumor and high grade prostate intraepithelial neoplasia (HGPIN) were confirmed;<br>(5) Tumor tissue images were reviewed by eight genitourinary pathologists, who reported the primary and secondary Gleason patterns of cancer for each slide and estimates of tumor cellularity in 10% increments (from 0%–100%).	The quality and quantity of samples do not meet any one or more of the criteria listed on the left.

Table 2 Cohort Characteristics

**Clinical Feature**	
Age	61 (43–76)
Preoperative PSA	7.4 (1.6–87.0)
NPTX2	5.8 (1.2–9.1)
**Gleason Score**	
3+3	65
3+4	102
4+3	78
≥ 8	88
**Tumor Cellularity (pathology)**	
<20%	7
21–40%	40
41–60%	84
61–80%	115
81–100%	87
**Pathologic Stage**	
pT2a/b	18
pT2c	111
pT3a	110
pT3b	82
pT4	6
**PSA Recurrence**	
Yes	33
No[a]	248
Not available	47
**Margin Status**	
Positive	69
Negative	193
Not available	71
**Ethnicity**	
Caucasian	270
African descent	43
Asian	8
Not available	12

[a] Either no evidence of recurrence or insufficient follow-up
